# Supplementary material for: Preparation and Application of Electrochemical Horseradish Peroxidase Sensor Based on a Black Phosphorene and Single-Walled Carbon Nanotubes Nanocomposite
Source: Molecules. 2022 Nov 20;27(22):8064. doi: 10.3390/molecules27228064 (PMC9694212; doi:10.3390/molecules27228064)
Supplement: Supplementary file 1 [file molecules-27-08064-s001.zip › molecules-1987812-supplementary.pdf]

Supplementary Information

Preparation and Application of Electrochemical Horseradish Peroxidase Sensor Based on a Black Phosphorene and Single-Walled Carbon Nanotubes Nanocomposite

Xiaoqing Li [1,2], Lisi Wang [1], Baoli Wang [1,3], Siyue Zhang [1], Meng Jiang [1], Wanting Fu [1] and Wei Sun [1,*]

1 Key Laboratory of Functional Materials and Photoelectrochemistry of Haikou, Key Laboratory of Water Pollution Treatment and Resource Rouse of Hainan Province, College of Chemistry and Chemical Engineering, Hainan Normal University, Haikou 571158, China

2 College of Health Sciences, Shandong University of Traditional Chinese Medicine, Jinan 250355, China

3 College of Health Sciences, Hainan Technology and Business College, Haikou 570102, China

* Correspondence: sunwei@hainnu.edu.cn

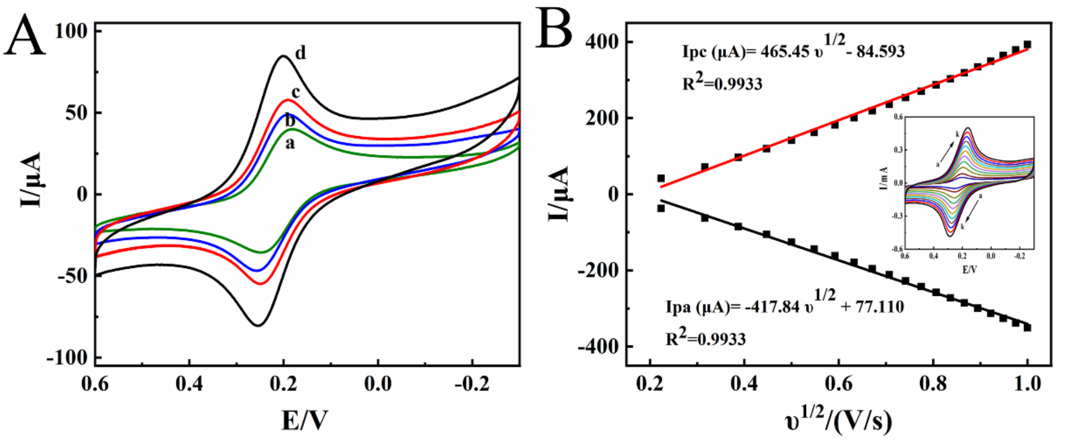

Figure S1. (**A**) Cyclic voltammograms of different electrodes (a) CILE, (b) Nafion/SWCNTs/CILE, (c) Nafion/BP/CILE and (d) Nafion/SWCNTs-BP/CILE in 1.0 mmol/L $K_3[Fe(CN)_6]$ and 0.5 mol/L KCl solution with scan rate of 100 mV/s. (**B**) Linear relationship of Ip with scan rate ($\upsilon^{1/2}$) (inset is influence of the scan rate on the electrochemical responses of Nafion/SWCNTs-BP/CILE, from a to k: 0.05, 0.1, 0.2, 0.3, 0.4, 0.5, 0.6, 0.7, 0.8, 0.9, 1.0 mV/s).
